# Protection from Small-Pox by Means of Vaccination and Re-Vaccination

**Published:** 1865-04

**Authors:** A. N. Bell, J. P. Loines, H. D. Bulkley, A. Nebinger, James F. Hibberd

**Affiliations:** Brooklyn, N. Y.; New York; New York; Philadelphia; Richmond, Ind.


THE
CHICAGO MEDICAL EXAMINER.

N. S. DAVIS, M.D., Editor.

VOL. VI. APRIL, 1865. NO. 4.

### Original Contributions.

### ARTICLE XII.

### PROTECTION FROM SMALL-POX BY MEANS OF VACCINATION AND RE-VACCINATION.

At the annual session of the American Medical Association, held in New York, June, 1864, the following resolutions were adopted, and the undersigned appointed a Committee to carry them out:—

"Resolved, That the Association deems it a duty to institute measures looking to the vaccination, ultimately, of every person living in the limits of country over which it exercises influence.

"Resolved, That a Central Committee of five be appointed to enlighten the public mind, by all available means, upon the value and necessity of universal vaccination.

"Resolved, That the Central Committee be authorized to appoint associate and auxiliary committees in each State."

According to the most reliable statistics obtainable, the annual death-rate of the United States is about one in forty-five of the whole population, or about 600,000 annually for the last ten years. Of this number of deaths from all causes, about one in every two hundred and fifty, or in round numbers, not less than 2,500 die annually of small-pox. This mortality, estimated by the usual ratio of deaths in small-pox, demonstrates the existence of more than 10,000 cases of unmodified small-pox annually in the United States, exclusive of varioloid; all of which can be wholly prevented by effectual vaccination and *re*-vaccination. During the great epidemics of small-pox which prevailed in Europe during the early part of the present century, all observers had abundant opportunities for ascertaining the true value of vaccination. The vaccinated, and those who had previously had small-pox, were both found to be more or less subject to the disease. Early in the progress of these epidemics, however, an important fact became evident, namely, that there was a great difference in the mortality of small-pox when it attacked these three classes:—1. The vaccinated; 2. The variolated; and, 3. Those who had neither been vaccinated nor had small-pox. Of the first class, those who had been previously vaccinated, out of every 330 attacked, only 1 died. of the second class, those who had previously had small-pox, 1 out of every 50 died. While of the third class, those who had neither been vaccinated nor had small-pox, 1 out of every 4 died. Thus proving the great superiority of vaccination over small-pox itself, in protecting the system from the fatality of a second attack.

Unmodified small-pox is not only a very fatal disease, as proved by its frequently cutting off one-fourth of all whom it attacks, but its tendency is to develope any latent disease which may exist in the constitution, particularly scrofula, and it is thus, indirectly, the cause of death to a much larger portion of the human race than at first sight appears. It is not, therefore, surprising that small-pox, attacking for the second time an already enfeebled constitution, should cause a greater proportion of deaths than takes place when it occurs after vaccination, a much milder form of the same disease, and, therefore, proportionately less likely to develope any pre-existing constitutional affection.

It may, indeed, be truly said, that during the last sixty-four years, vaccination has not only prevented millions of cases of small-pox, but that it has also saved a multitude of persons from scrofula and other fatal maladies.

The human race has become stronger, has produced more hardy offspring;—not only suffering less from disease in general, but in all respects more capable of resisting it, through the instrumentality of vaccination.

Among the earliest objections urged against vaccination, even during the time of JENNER, was the alleged danger of communicating other diseases with the vaccinia. And from that day to this, cases of cutaneous disease, syphilis, scrofula, &c., have been occasionally attributed to this cause. But if it were now possible to collect all such cases, even then, their utter insignificance when compared with the multitude that have unknowingly been vaccinated with lymph taken from diseased persons, without contamination, is such, that this evidence alone against the transmissibility of other diseases by vaccination, would be sufficient to establish the conclusion as a general rule, that *there is no danger of communicating other diseases with vaccinia.* This conclusion is in keeping with the recorded experience of HEIM, RICORD, BOSQUET, TAUPIN, LANDOURY, FRIEDINGER, and many others, who have investigated the subject, and may, indeed, be regarded as an accepted truth by the most distinguished men of the medical profession.

The recorded facts, the arguments, and deductions made by investigators in reference to the possibility of transmitting other diseases with vaccinia, added to the facts positive and negative which have presented under our own observation, not only confirm our belief in the non-transmissibility of other diseases with the vaccinia *as a general law,* but demonstrate that vaccination, besides preventing small-pox, frequently fortifies the constitution of the individual against some of the worst of those very diseases which are erroneously alleged to be transmitted by it. We would not, however, be understood as approving of the use of vaccine virus obtained from diseased persons; but on the contrary—*none should ever be used knowingly, except that which has been obtained from perfectly healthy subjects.*

The protective powers of vaccination are most evident in their results to mankind in general. The comparative exemption of all civilized communities, for the last forty years, from the most terrible scourge to which mankind was ever liable, is an evidence of the protection afforded by vaccination so overwhelming as to characterize the discovery of JENNER as the greatest boon ever conferred upon the temporal welfare of man.

In England alone, for nearly a century before the introduction of vaccination, no fewer than 30,000 persons were annually cut off by small-pox; which, in the same ratio, according to the present population, would be equivalent to 100,000 deaths annually. Out of every 1,000 deaths, from 1750 to 1800, there were by small-pox 96; from 1800 to 1850, there were, for the same number, but 35.

In Germany, for the same periods of time, there were, out of every 1,000 deaths for the former period, by small-pox 66.5; for the latter period, 7.26.

In Sweden, for the last twenty-eight years before the introduction of vaccination, out of each million of the population there were 2050 deaths annually by small-pox; for forty years subsequent to the introduction of vaccination, the number of deaths annually by small-pox, per million of inhabitants, was 158.

In Westphalia, from 1776 to 1780, the annual small-pox death-rate per million of inhabitants, was 2643. From 1816 to 1850, it was 114.

In Copenhagen, 1751 to 1800, the annual rate per million was 3128; from 1800 to 1850, it was 286. In Berlin, for the same periods, the rates relatively were 3442, and 176.

American statistics, so far as known, are equally conclusive. According to a paper read by Dr. ROBERT WARE, before the Boston Sanitary Association, we learn that in Boston, in 1721, the year in which inoculation was introduced, and when the whole population was 11,000, 5759, or more than one-half had small-pox, and of these, 844 died. In 1730, there were 4000 cases and 200 deaths. In 1752, when the population was 15,684, there were 5545 cases, and 539 deaths. In 1764, there were 5646 cases; in 1776, 5292; and in 1792, 8346. Compared with a subsequent period, after the general introduction of vaccination, and when it was in a measure compulsory, from

1815 to 1830, the mortality from small-pox was only *fourteen;* from 1811 to 1839, it was but *fifty-two.*

By an approximate average death-rate by small-pox per million of living population, from 1750 to 1800, and from 1800 to 1850, in various countries (collected by the Epidemiological Society of London), there were, in the former period, nearly twenty times as many deaths by small-pox as there were in the latter. And besides, "one obviously beneficial result of vaccination, as regards its protective influence on the multitude, has not, we think, been appreciated or illustrated with sufficient force; namely, that the epidemic influence of small-pox greatly increased during the practice of inoculation, and greatly decreased since vaccination has been adopted. Dr. HEBRA, Professor of Diseases of the Skin, at Vienna, incidentally remarks and simply alludes to the fact, that 'Epidemics of small-pox have been more rare, and are less malignant, since the introduction of vaccination.' But definite material for the following statements are to be found in the report of the Epidemiological Society, in illustration of our remarks:—(1.) During 91 years previous to *inoculation*, there were 65 distinct and well-marked epidemics, which is a ratio of 71.4 epidemics in 100 years. (2.) During 63 years, in which *inoculation* was practiced, and that to a great extent, there were 53 distinct and well-marked epidemics,which is a ratio of 84 epidemics in 100 years. (3.) During the last 50 years, in which vaccination has been practiced, and inoculation been declared illegal, there have been 12 epidemics of small-pox, which is a ratio of 24 epidemics in 100 years."*

According to the well kept mortality statistics of Sweden, the *annual* small-pox death-rate in that country, during the period of 1841–50, averaged less than the *weekly* death-rate from small-pox and measles during the period of 1755–75. This important fact introduces us to an indirect benefit of vaccination, which has, until recently, been overlooked, namely, the beneficial influence of vaccination against other diseases. Drs. GREENHOW and FARR, under the auspices of the General Board of Health of London, have shown that with the decline of small-

* Medico-Chir. Review, October, 1857.

pox consequent on vaccination, the general death-rate has greatly diminished from all causes; and that too, notwithstanding a severe and fatal epidemic of influenza and two epidemics of cholera; and under this diminution, it is especially notable that the two classes of disease usually considered the most fatal, namely, scrofulous and low febrile affections, have diminished in a remarkable degree. The general death-rate per 10,000 of living population, during the periods of 1846–55, was 25 per cent. less than the decennial period of 1746–55; and 40 per cent. less than the decennial period of 1681–90, showing a successive decline since the remoter period, from 421 to 355; and since the more recent period, from 355 to 249.

According to Dr. FARR'S statistics, the average annual death-rates in London, from all causes and all ages, per 10,000 living were:—

| | |
|---|---|
| From 1771–80 | 500 |
| 1801–10 | 292 |
| 1831–35 (small-pox prevailed) | 320 |
| 1840–54 | $248\frac{9}{10}$ |

The average annual death-rates in Sweden, from all causes and all ages, per 10,000 living were:—

| | |
|---|---|
| From 1776–95 | 268 |
| 1821–40 | 233 |
| 1841–50 | 205 |

In McCulloch's Descriptive and Statistical Account of the British Empire, Dr. FARR has shown that fever has progressively subsided since 1771, (at first under the influence of innocculation); "and that *the combined mortality of small-pox, measles, and scarlatina is now only half as great as the mortality formerly occasioned by small-pox alone.*"

According to the researches of Dr. GREENHOW, previous to the introduction of vaccination, the death-rate from scrofulous diseases was five times greater than it is at the present time; and the present death-rate of pulmonary consumption, great as is, is seven per cent. lower than it was previous to the discovery of JENNER.

M. Bousquet,* in his detail of the epidemic which prevailed at Marseilles, in 1825, states that the whole population was estimated at 40,000. Of these, 30,000 had been vaccinated; 2000 had had small-pox, 8000 had neither been vaccinated nor had small-pox. Of the 30,000 vaccinated, 2000 were seized with small-pox; 20 of whom, or one for every hundred affected, died. Of the 2000 who had before had small-pox, either naturally or by inoculation, 20 were attacked, and of these 4 died, or one for every five who took the disease. Of the 8000 who had not been vaccinated nor had small-pox, 4000 contracted it, and 1000 died, or one in every four. By this it appears that *one-half* of the non-vaccinated, *one-fifteenth* of the vaccinated, and *one one-hundreth* of the variolated took the disease. But such was the difference in the comparative mortality of the attack in the vaccinated and the variolated, that while the variolated part of the population were cut off in the proportion of *one* out of every 500, the vaccinated only lost *one* out of every 1,500; or, in other words, of an equal number of variolated and vaccinated cases, *three* of the variolated died from the second attack, for every *one* that died who had been previously vaccinated.

Re-Vaccination.—Several governments, confident in the anti-variolous power of vaccination, and profiting by the experince gained, determined upon re-vaccination as the most likely means of getting rid of the epidemics which were desolating their armies. The effect was so salutary as to have finally banished small-pox from among them. But this was not the only benefit. Knowing, as we do, that small-pox and cow-pox are in reality the same disease, the latter being merely deprived of its virulence by having previously passed through the system of the cow, the results of these numerous re-vaccinations are of immense importance not only in confirming the identity of small-pox and cow-pox, but in establishing the no less important fact, that the protective power of small-pox itself wears out of the system in a certain proportion of cases, as life advances, in nearly the same ratio as that of cow-pox. Thus, in all these

* Traite de la Vaccine.

armies, a certain proportion of the men were found to have been previously vaccinated, while no inconsiderable proportion had passed through unmodified small-pox. Now, if we take the susceptibility to re-vaccination as a test of liability to varioloid or to a second attack of small-pox, we have these vaccinations proving the fact that after a certain number of years, the same proportion of those who have previously had small-pox become susceptible to a second attack, as those who have been vaccinated are to varioloid. So that once having passed through all the dangers of unmodified small-pox, the person, at the end of twenty years, for instance, has no better security against a second attack, than the person who has been vaccinated for a corresponding length of time.

In the Wurtemberg army, of 40,000 cases collected by Dr. Heim, on re-vaccination it was found that in every 100 vaccinated after small-pox, 32 succeeded, 26 were modified, and 42 failed. In every 100 re-vaccinated 34 succeeded, 25 were modified, and 41 failed.

According to the Statistical Report of the Medical Department of the English Army, from Oct. 1858, to Dec. 1859, 32,510 soldiers and recruits were vaccinated. Of this number, 4124 bore marks of unmodified small-pox; 23,924 bore good marks of vaccination; 1901 bore doubtful marks of vaccination; and 2561, had no marks of either vaccination or small-pox.

By reducing these figures as nearly as possible to the same scale as those above given for the army of Wurtemberg, we find that of the 4124 who had previously had small-pox, on vaccination, 1473 or 35 per cent. succeeded; 799 or 19 per cent. took imperfectly; and 1842 or 44 per cent. failed. Of the 23,924 who had good marks of vaccination, on re-vaccination 8976 or 37 per cent. took perfectly; 5277 or 22 per cent. imperfectly; and 9671 or 40 per cent. failed. Of the 1901 with doubtful marks of vaccination, the number of good vesicles on re-vaccination was 777 or 40 per cent.; modified, 505 or 26 per cent.; and 616 or 32 per cent. failed. Of the 2561 who had no evidence of protection, on vaccination, 1362 or 53 per cent.

took; 484 or 18 per cent. had modified vesicles, and 715 or 23 per cent. failed.

In the Prussian Army, in 1860, 69,096 soldiers were vaccinated. Of this number 57,325 exhibited marks, more or less perfect, of previous vaccination, 7420 being distinct; 4151 showed no marks whatever. Of the whole number, 44,193 took; 8256 partially and 16,047 failed. These last were vaccinated again, when 5577 proved successful, and 11,650 failed. During the year, there occurred among those who had been unsuccessfully vaccinated and others who had been successfully vaccinated in former years, one case of varioloid, but no case of small-pox. Thus, during the year 1860, out of 69,096 vaccinations, 49,777 or 72 per cent. took. In the entire army, there occurred during the year 23 cases of varioloid, and 4 of small-pox. Of these cases, 14 of varioloid and 4 of small-pox occurred in persons who had not been re-vaccinated; 8 of varioloid, and 1 of small-pox, occurred among those in whom the re-vaccination failed; and the remaining 1 of varioloid, among those who had been re-vaccinated with success.

In the United States, re-vaccination has received but little attention. On the breaking out of small-pox among a crew of five hundred persons, on board a U.S. frigate, about twenty-five years ago, the late Dr. Samuel Jackson, Surgeon U.S.N., vaccinated the whole ship's company. One in six took. They had all been vaccinated or had had small-pox before. Those on whom vaccination failed, proved to be equally insusceptible to small-pox, which wholly ceased from the time the re-vaccinations took effect.

Of 686 recruits vaccinated by Dr. Forry, U.S. Army,* 560 had been vaccinated before, 74 had had small-pox, and 52 had not had small-pox nor been vaccinated. Of the 560 previously vaccinated, 381 exhibited good cicatrices; 134 imperfect, and 45 had no marks at all; 196 took, including 55 which were modified. Of these 196, 109 had been previously vaccinated before the age of five years; 48 between the ages of five and ten, and 39 subsequently to the latter age. Of the whole 560 pre-

* American Jour. Med. Sciences, April, 1842.

viously vaccinated, 316 were vaccinated before the age of five years; 133 between the ages of five and ten, and 111 after the latter period. Hence it follows, though not as an exact result, according to Dr. FORRY'S limited experience, that as the ages of the great majority of these men ranged from twenty to thirty-three (the average being twenty-five), and as the ratio of successful re-vaccination is very nearly the same after each interval of age (being about one-third), the limit of the protective power of vaccination is not restricted to any precise number of years.

The only statistics of re-vaccination of the present army of the United States, we have been able to obtain, are the following, furnished by Dr. S. O. VANDERPOEL, Surgeon-General of New York, to the U.S. Sanitary Commission,* from the first returns made to him in accordance with a general order:—

| | |
|---|---|
| Total number of recruits examined | 9548 |
| Bearing marks of previous vaccination | 7765 |
| Total vaccinated or re-vaccinated | 8095 |
| Found to be susceptible | 2292 |
| No. susceptible having marks of previous vaccination | 1338 |

It is evident from the foregoing statistics, that no certain period of limitation can be fixed for the protective power of vaccination. It is certain, however, that its loss of power bears *some* proportion to the lapse of time, though it seems highly probable that this apparent loss of protective power is in the same ratio as the varying liability to small-pox, independent of vaccination. Dr. J. F. MARSON, the experienced superintendent of the small-pox and vaccination hospital in London, states that, "but few patients under ten years of age have been received with small-pox after vaccination. After ten years, the number begins to increase considerably, and the largest number admitted are for the decennial period from the age of fifteen to twenty-five, and although progressively diminishing, they continue rather large up to thirty, and from thirty to thirty-five they are nearly the same as from ten to fifteen; but as in the unprotected at this period of life, the mortality is doubled, showing the cause to be probably as much or more

* Sanitary Commission Report, E.

depending on age and its concomitants as on other circumstances. In still further advanced life, the ratio of mortality will be seen to increase also, as in the unprotected state."*

According to the statistics of Professors HEIM, of Stuttgart, and RETZIUS, of Stockholm, and Dr. MARSON, of London, the liability of small-pox is found to be, as regards age, very nearly the same as the increased susceptibility to a second vaccination, or, as will presently be seen, to a second attack of small-pox.

The occasional recurrence of unmodified small-pox a second time, or after a previous vaccination, does not invalidate the general law, that a person who has once been properly vaccinated, or has once had small-pox, in general remains protected against a subsequent attack. It is, however, a well established fact, that certain individuals who have had unmodified small-pox in infancy or youth, may, especially if frequently exposed to the epidemic influence of the disease, have it again in after life; and such attacks are always much more dangerous to life than small-pox after vaccination. All medical men of much experience have met with such cases. Dr. THOMPSON, of Edinburgh, in his own practice, met with 85 cases of second attack of unmodified small-pox; and Prof. HEIM, 57. It is in vain, therefore, to expect that vaccination will give greater security to the person from a subsequent attack of small-pox than small-pox itself unmodified. All that can be reasonably asked is, that vaccination shall give as good security against a subsequent attack of small-pox as if the person had passed through small-pox itself; and this, if properly performed, and with good lymph, the accumulated evidence of the last sixty years most thoroughly proves. And with this immense superiority in favor of the protective power of vaccination over unmodified small-pox, namely:—that it is not contagious; does not endanger life; does not engender scrofulous disease; does not disfigure the countenance, nor cause deafness and blindness, and does not cut off one in every four to eight affected by it—with all of which small-pox is justly chargeable.

* Returns of Epidemiological Society, London.

By estimating the result of the foregoing statistics (chiefly obtained from the returns of the Epidemiological Society of London, and more might be furnished), embracing carefully recorded and reconcilable points of observation for nearly 200,000 cases, we are justified in accepting this experience as a safe criterion by which to base an estimate for any number of cases, great or small.

The conclusions elucidated from the data given, are:—

1. That vaccination is immensely protective against epidemic diseases generally, and against small-pox in particular; and against death by small-pox, the protective power of vaccination is almost perfect.

2. That of any number of persons who have had unmodified small-pox, the proportion wholly protected from a second attack at adult age, is 43 per cent., while 57 per cent. are liable to it again in some form or other.

3. That out of any number of adult persons who have good marks of vaccination, 40½ per cent. are perfectly protected; while 59½ per cent. are susceptible to varioloid, or to re-vaccination, to such a degree as to render their protection perfectly complete.

4. That the degree of protection afforded by previous unmodified small-pox, from a second attack, is only 2½ per cent. greater than the protection afforded by vaccination; a proportion too small to be regarded as any evidence of real difference in protective power, and reasonably attributable to spurious or impaired vaccination from a variety of causes, such as vaccination during the progress of other diseases, injury of the vesicle, or defective lymph.

5. That out of any number of adult persons with imperfect marks of vaccination, 23 per cent. only are protected, while 77 per cent. are liable to small-pox or varioloid.

6. The liability to varioloid after ten years of age, of persons vaccinated under three years of age; and the increased liability again from fifteen to twenty-five years of age, of persons vaccinated or re-vaccinated at from ten to fifteen years of age, demonstrates that, generally, protection by vaccination under twenty-five years of age is complete for about seven years only. Subsequent to twenty-five years of age, protection is complete for a greater length of time, proportionate to the age of the individual at the time of the vaccination.

7. That during the prevalence of an epidemic of small-pox there is increased susceptibility to the disease, and the degree of protection from previous vaccination is proportionately lessened. Under such circumstances, therefore, it is incumbent to re-vaccinate at intervals not exceeding five years; and in cases of certain exposure, as soon as practicable thereafter, as there is abundant evidence of the protective power of vaccination and re-vaccination even as late as the fourth day after exposure to small-pox.

8. Protection is known to be complete only when fresh vaccine lymph from a perfect visicle fails to take on re-vaccination.

A. N. BELL, *Brooklyn, N.Y.*
J. P. LOINES, *New York.*
H. D. BULKLEY, *New York.*
A. NEBINGER, *Philadelphia.*
JAMES F. HIBBERD, *Richmond, Ind.*

